# The effects of distraction on metacognition and metacognition on distraction: evidence from recognition memory

**DOI:** 10.3389/fpsyg.2014.00439

**Published:** 2014-05-14

**Authors:** C. Philip Beaman, Maciej Hanczakowski, Dylan M. Jones

**Affiliations:** ^1^Centre for Cognition Research, School of Psychology and Clinical Language Sciences, University of ReadingReading, UK; ^2^School of Psychology, Cardiff UniversityCardiff, UK

**Keywords:** metacognition, memory, recognition, auditory distraction, irrelevant speech

## Abstract

The effects of auditory distraction in memory tasks have, to date, been examined with procedures that minimize participants’ control over their own memory processes. Surprisingly little attention has been paid to metacognitive control factors which might affect memory performance. In this study, we investigate the effects of auditory distraction on metacognitive control of memory, examining the effects of auditory distraction in recognition tasks utilizing the metacognitive framework of Koriat and Goldsmith (1996), to determine whether strategic regulation of memory accuracy is impacted by auditory distraction. Results replicated previous findings in showing that auditory distraction impairs memory performance in tasks minimizing participants’ metacognitive control (forced-report test). However, the results revealed also that when metacognitive control is allowed (free-report tests), auditory distraction impacts upon a range of metacognitive indices. In the present study, auditory distraction undermined accuracy of metacognitive monitoring (resolution), reduced confidence in responses provided and, correspondingly, increased participants’ propensity to withhold responses in free-report recognition. Crucially, changes in metacognitive processes were related to impairment in free-report recognition performance, as the use of the “don’t know” option under distraction led to a reduction in the number of correct responses volunteered in free-report tests. Overall, the present results show how auditory distraction exerts its influence on memory performance via both memory and metamemory processes.

## INTRODUCTION

Distraction, whether in the form of external stimuli or self-generated thoughts, accompanies a vast spectrum of our everyday activities. Much of this can be avoided by relatively simple actions, like closing one’s eyes if the distraction is visual, but some forms of distraction cannot be done away with so easily. Auditory distraction in particular is impossible to avoid unless we have control over the source of distraction, or else access to noise-reduction technology (e.g., headsets). If we do not have such control, for example, when we are in a supermarket and music plays over the store’s loudspeakers, our cognitive processes need to unfold in the presence of distraction. This can constitute a serious problem inasmuch as numerous studies have found that the efficacy of cognitive processes suffers in the presence of auditory distraction (see reviews by Hughes and Jones, 2003; Beaman, 2005a; Jones et al., 2010). Most relevantly to the purpose of the present study, decades of studies of memory processes have found that auditory distraction present either at encoding or retrieval negatively impacts upon memory performance (e.g., Broadbent, 1982; Salamé and Baddeley, 1982, 1986; Miles et al., 1991; Jones and Macken, 1993; Elliott and Cowan, 2005; Bell et al., 2013).

Although the negative impact of auditory distraction upon memory performance is well-documented, what still remains unexplored is how people strive to adapt to auditory distraction they cannot avoid. Recent developments in theoretical approaches to memory processes stress that memory processes are far from passive, rather they are subject to a number of control operations. A metacognitive approach to memory describes how people monitor their memory performance under a variety of conditions and how the products of metacognitive monitoring are employed in an attempt to optimize memory performance (see Koriat, 2007, for a review). Thus, for example, people try to establish whether encoding of information is satisfactory and, whenever this process of monitoring informs them that certain information is poorly learned, additional study time may be allocated to this information (Thiede and Dunlosky, 1999). Similarly, during retrieval people monitor whether retrieved information is likely to be correct and whenever this process of monitoring informs them that certain information may be incorrect people may choose to withhold it from a memory report (Koriat and Goldsmith, 1996; Higham, 2007). The question that we begin to address here is how the processes of metacognitive monitoring and control at test are affected by, and feed back onto, auditory distraction. Suppose effective metacognitive monitoring and control allows individuals to compensate for the impact of distraction (interpreted here as an outcome). Discovering the circumstances under which this is possible would demonstrate the effects of metacognition on distraction and would constitute a practical as well as a theoretical advance. Alternatively, suppose that effective metacognitive monitoring and control becomes more difficult when distracted (i.e., being in the *state* of distraction), the monitoring of output may be disturbed as much as the initial encoding of items in a memory task, for example. This would demonstrate an impact of distraction on metacognition.

**FIGURE 1:**
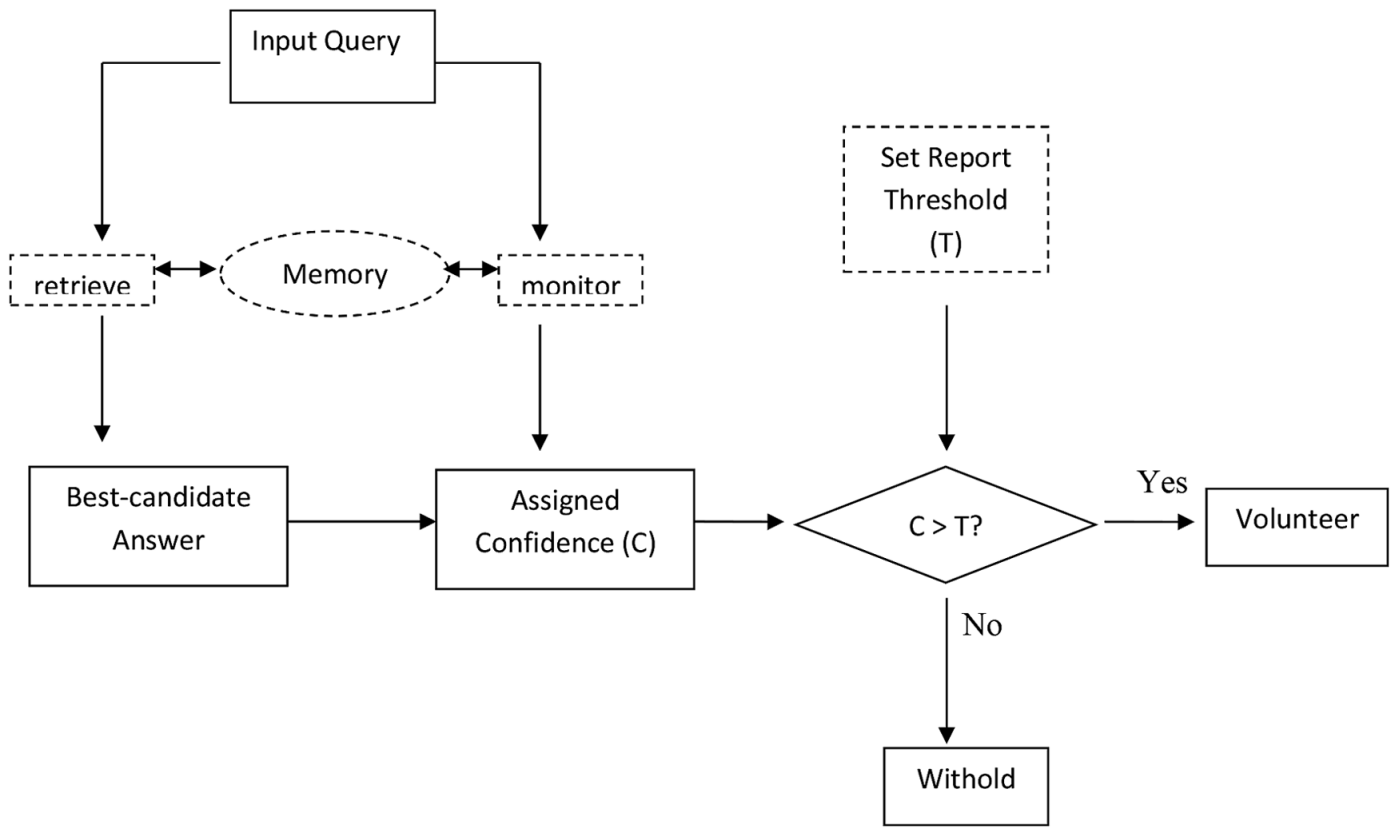
**How distraction might affect metacognition and metacognition might affect distraction.** Adapted from Goldsmith et al. (2014). Dashed boxes represent operations which might be affected by distraction. Memory, its retrieval or monitoring processes might be disrupted by distraction, and the meta-cognitive process of setting a response threshold might mediate the impact of distraction. Note that if distraction lowers confidence in an output, then this will have the same negative effect on volunteering the item as raising the report threshold for that output.

To describe how people monitor the accuracy of the products of retrieval processes and how they exert metacognitive control, Koriat and Goldsmith (1996) developed a framework within which to examine the decisions made as to whether retrieved information should be volunteered or withheld from a memory report. In this framework it is assumed that responding to a memory question unfolds in three steps. At the first step, a person accesses memory to generate the best, or most likely, candidate response to the question. In the next step, the person monitors the retrieval process, assigning confidence that his/her best candidate response is correct [assigning confidence in this way may be either a strategic or a wholly unconscious process – we are ambivalent on this point. Koriat and Goldsmith (1996) use the more strategic-sounding term “assessing probability” to describe this process but there is no necessary assumption that the person is making subjective probability judgments either consciously or deliberately, (see also Goldsmith et al., 2014)]. Finally, in the third step, the person compares the confidence for a given candidate response to a criterial or threshold level of confidence which warrants volunteering of candidate responses in a memory report. If confidence in the correctness of a given candidate response is higher than criterion, this candidate response is volunteered. However, if confidence in the correctness of this candidate response is lower than criterion, it is withheld and the individual responds “don’t know” to the memory question (see **Figure 1**).

Crucially, this framework postulates that, whenever withholding responses is allowed, the ultimate memory performance observed is jointly shaped by memory processes responsible for generating candidate responses and metacognitive processes responsible for deciding which candidate responses should be volunteered. Koriat and Goldsmith (1996) describe two different indices of memory performance. The input-bound accuracy (IBA) measure refers the number of correct responses in a test relative to the number of questions asked (which is usually equivalent to the number of items presented for study). Essentially, it is a measure of the *quantity* of correct information volunteered at output. Thus, for example, if persons A and B are given 20 questions and respond correctly to five of these, then the IBA measure for both these individuals is 25%. The output-bound accuracy (OBA) measure refers the number of correct responses in a test relative to the overall information that a person provides in this test. Essentially, it is a measure of the *quality* of memory output. Thus, if A declines to answer 15/20 questions then A’s OBA is 100%, whereas if B responds incorrectly to five of the questions, then B’s OBA measure is 50%. Although in a forced-report test IBA and OBA measures are necessarily identical, in free-report they become different measures of memory performance, sensitive to changes in metacognitive processes.

In their seminal paper, Koriat and Goldsmith (1996) described several scenarios by which changes in memory and/or metacognitive processes affect IBA and OBA measures. Changes in underlying memory have quite straightforward consequences: better memory should be linked to increased correct responding, whether measured in reference to the volume of studied information (the IBA measure) or to the volume of output information (the OBA measure). However, the consequences of changes in metacognitive processes for memory performance are less straightforward. Broadly speaking, people can modify performance by varying the criterial value of confidence, which is then captured by IBA and OBA measures in different ways. If people want to increase the quantity of correct information provided in their memory reports they can lower the criterion, ensuring that more candidate responses achieve or exceed this criterion. This should generally result in an increase in the IBA measure. However, because the ability to distinguish between correct and incorrect candidate responses is almost always going to be less than perfect, lowering the criterion can also result in volunteering some incorrect candidate responses. In fact, the more candidate responses are volunteered, the poorer the quality of these additionally volunteered responses should normally be, leading to a reduction in the OBA measure. Consequently, the IBA and OBA measures are subjected to a trade-off: an increase in the quantity obtained by lowering report criterion should generally be accompanied by a reduction in the quality and, similarly, an increase in the quality obtained by adopting a more stringent criterion should generally be accompanied by a reduction in the quantity of correct answers volunteered in a memory report.

Currently, little is known about how auditory distraction impacts upon metacognitive regulation of memory responses as captured by the Koriat and Goldsmith (1996) framework. The majority of studies on auditory distraction have used either free or serial recall tests which allow participants to withhold answers and respond “don’t know.” Such tests do not allow for disentangling memory and metacognitive effects of distraction because omissions from a memory report can reflect either a failure to access an appropriate memory trace or a change in metacognitive processing in one or more ways. For example, participants may become less confident of their candidate responses, so that fewer of them pass the report criterion. Alternatively, participants may become more cautious and adopt a more stringent report criterion (see **Figure 1**). To assess any presumed metacognitive component of distraction it is first necessary to present participants with a test (such as recognition) in which responding is commonly forced, not allowing participants to respond “don’t know.” Whilst this is comparatively rare in studies of auditory distraction, a sub-set of studies have employed such recognition memory tests. For example, Beaman and Jones (1997) investigated the effects of auditory distraction (sequences of non-sense words which participants were asked to ignore played over headphones) on a two-alternative forced-choice (2AFC) recognition test and found reliable impairment in recognition performance in the distraction condition (see also LeCompte, 1994; Stokes and Arnell, 2012). Since performance in forced recognition tests is not dependent on metacognitive processes of response withholding, this result confirms that auditory distraction impairs memory access directly.

The outstanding question then remains whether impairment to memory by auditory distraction is also accompanied by changes in metacognitive processes, or whether the distraction is limited to memory. For example, do people try to compensate for the impairment caused by distraction? It is possible that knowing that distraction impairs memory access, participants could change their report criterion in order to compensate for distraction and volunteer responses held with lower confidence, thus increasing the quantity of output. Indeed, a recent study by Perfect et al. (2011) examined the effects of distraction at retrieval and eye-closure (as a strategic response) on memory for actions and found that distraction did not reduce correct responding but instead increased the number of incorrect responses (an effect partially mitigated by eye closure). As noted by Perfect et al. (2011) such a pattern of results is most easily explained if distraction impairs memory and participants react to this impairment by adopting a more liberal report criterion, thus volunteering candidate responses held with lower confidence. In other words, participants in this study could strive to increase the IBA measure of memory performance, sacrificing the OBA measure in the process.

It should be also noted, however, that results obtained by Perfect et al. (2011) are atypical for the auditory distraction of the type briefly reviewed here, which more usually reduces the number of correct responses volunteered. This is particularly noticeable in free recall where the difference between “irrelevant speech” and quiet conditions is often evident only in the number of correct items volunteered (where there are typically few or no incorrect items in the recall protocol – the exception being when the irrelevant speech is semantically related to the to-be-recalled items and intrusions from the speech into the recall protocol then become relatively common; see Beaman, 2004; Beaman et al., 2013). This common result may suggest that participants typically do not become more liberal in their reporting strategies under distraction. Such a conclusion may, however, be premature. If distraction impairs memory, it may also impair the resolution of metacognitive monitoring, that is, people’s ability to distinguish between their correct and incorrect candidate responses.

As described by Koriat and Goldsmith (1996), the ability to regulate memory performance, crucially depends on the resolution of metacognitive monitoring. The worse people are in distinguishing between correct and incorrect candidate responses, the less efficient their attempts to increase IBA will be. When participants decide to volunteer information held with lower confidence but resolution of their monitoring is poor, a substantial proportion of additionally volunteered responses will be incorrect, exerting little influence on IBA while at the same time undermining the OBA measure of memory performance. If auditory distraction were to impair the resolution of metacognitive monitoring at retrieval, then this could render any participants’ attempts to increase the quantity of correct information in a memory report futile. Worse still, it could degrade their memory performance further by increasing the rate of intrusions. However, the extent to which resolution of metacognitive monitoring is affected by auditory distraction is currently not known.

The present study was designed to examine the effects of auditory distraction on both memory and metacognitive monitoring and control of retrieval. To this purpose, we used a procedure in which participants studied and were tested on pairs of unrelated words, with both study and test phases performed either in silence or under conditions of auditory distraction. The tests we used were 2AFC recognition tests, in which participants were asked to discriminate between a target pair and a foil pair (the types of targets and distracters used in the study are described later). Crucially, the recognition tests were specifically designed to assess both memory and metacognitive processes. Each trial of the test consisted of three separate steps (see Hanczakowski et al., 2013, for this type of a testing procedure). In an initial free-report step, participants were instructed to preserve the quality of their reports and to endorse one of the alternatives only if they were sure it was correct, while responding “don’t know” if they were not sure. In the subsequent forced-report step, the “don’t know” option was no longer available and participants were asked to endorse one of the alternatives even if it required guessing. Finally, in the third step, participants were asked to provide a confidence judgment regarding the accuracy of a decision they made during forced-report.

This procedure allows for describing participants’ behavior in terms of the concepts developed in the Koriat and Goldsmith (1996) framework. The data from the initial free-report step allows for examining which of the candidate responses are volunteered. In conjunction with confidence judgments obtained for volunteered and withheld responses, this gives a basis upon which to assess the report criterion participants adopt in various conditions (e.g., under distraction). The forced-report test meanwhile serves as a relatively pure measure of the accuracy of direct memory access. Finally, the confidence judgments collected for responses provided in the forced-report step allow for examining the resolution of metacognitive monitoring, i.e., participants’ ability to distinguish between their correct and incorrect candidate responses. This framework was employed to assess the impact of auditory distraction on memory and metacognitive processes. Specifically, we were interested: (1) whether distraction impairs memory access, as assessed in the forced-report test (a direct effect of distraction on cognition), (2) whether distraction impairs the resolution of metacognitive monitoring (an effect of distraction on a metacognitive process), (3) whether participants modify their report criterion under distraction (metacognition thereby having a modifying effect on the observed impact of distraction), and (4) how any possible changes in memory and metacognitive processes caused by distraction are reflected in the IBA and OBA measures of memory performance.

Apart from manipulating the presence of distraction at study and test, we also manipulated the nature of the recognition test. The manipulation of the type of test was introduced to examine the impact of auditory distraction on memory and metacognition under testing conditions varying in the contribution of controlled retrieval processes required. A recent investigation of auditory distraction revealed that negative effects of distraction on recognition performance were confined to conditions that require controlled retrieval, such as retrieval of contextual details, and may not be revealed in simple old/new judgments that can be made based on familiarity (Wais and Gazzaley, 2011). In our study, each list of pairs of words participants studied was followed by three separate tests, each using one third of the studied pairs as a source of targets and each presented in the three-step format described above. The first two tests were a 2AFC *associative* recognition test and a *simple* 2AFC recognition test. In the 2AFC associative recognition test foils were pairs composed of two previously studied words recombined to create a novel pair. It was not possible to succeed in this test by identifying one or both of the words as unfamiliar (as both had previously been encountered in the experimental context). Instead, participants needed to recollect which pairs had previously appeared in which combination. In the simple 2AFC recognition test targets were previously studied pairs and foils were always composed of two novel words. In this test, participants could succeed if they if they recognized the target (successful identification of the target) or if they identified either or both of the words as unfamiliar (successful rejection of a foil). The results obtained by Wais and Gazzaley (2011) lead to the prediction that the effects of auditory distraction should occur in the associative test, which relies on recollection, but not in the simple test, which can be completed with the use of familiarity.

**FIGURE 2:**
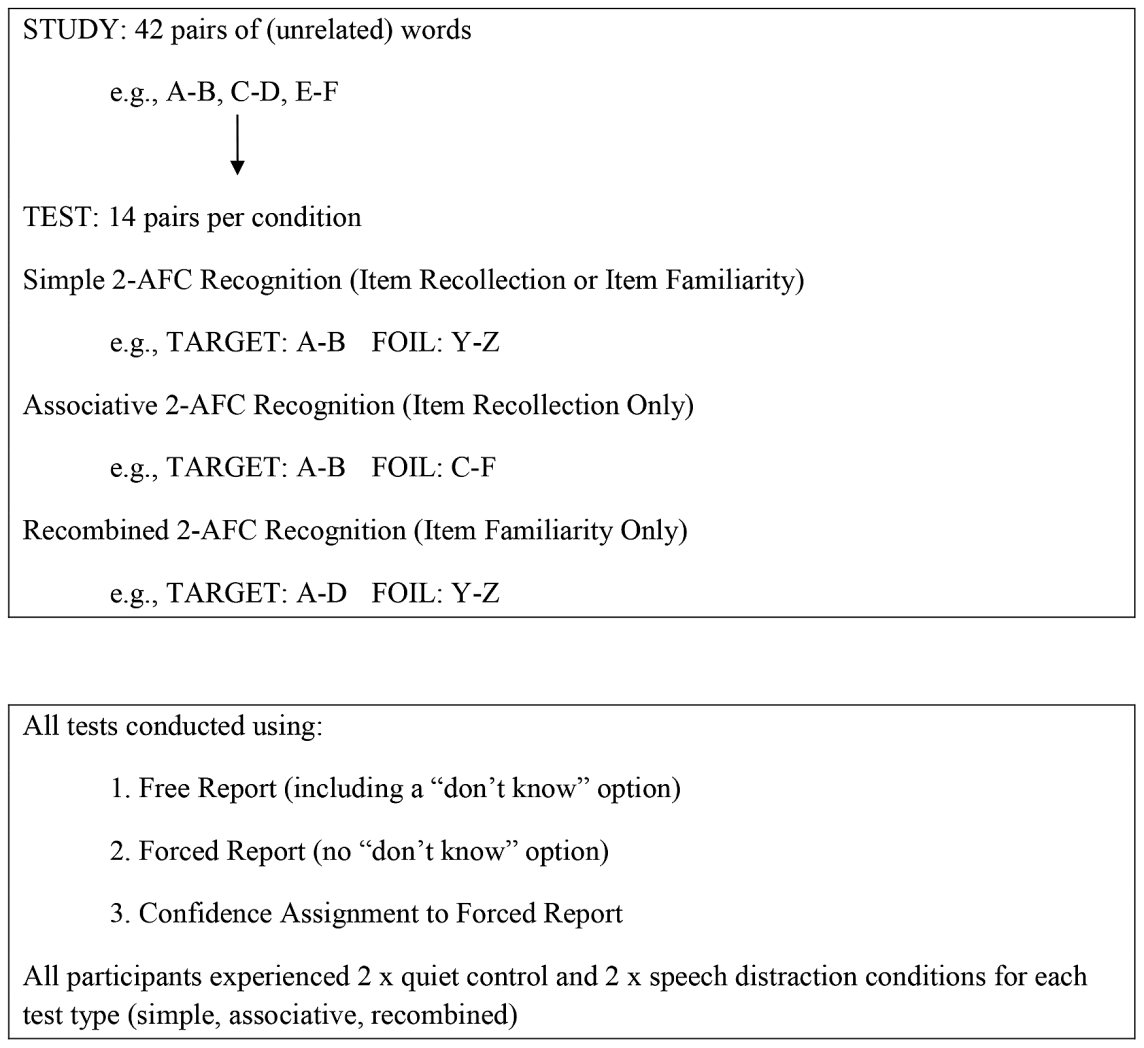
**Representation of the experimental design**.

It is important to note that, although simple recognition can be completed with the use of familiarity, recollection may still contribute to performance because reinstating an intact study pair at test may cue the pairwise association established between words at study (Cohn and Moscovitch, 2007). To provide a stronger test of the idea that the effects of auditory distraction are confined to recollection, we included a third type of test. The third test was a *recombined* 2AFC recognition test in which foils were again composed of two novel words (as in the simple test) but targets were pairs composed of recombined words, which were words included at study in different pairs. In this recombined test participants were asked to endorse pairs composed of two studied words, regardless of whether these pairs had originally appeared together. Because the original word–word association that could serve as a memory cue is not reinstated at test for the recombined pairs, the contribution of recollection to performance in this test should be further reduced. Thus, if auditory distraction impacts upon recollection only, it should have minimal impact upon this recombined test. A graphical example of the testing procedure is given in **Figure 2**.

## MATERIALS AND METHODS

### PARTICIPANTS

Forty-two undergraduates from Cardiff University (mean age = 21.66, range 18–40, 5 males) participated for course credit or small monetary compensation.

### MATERIALS AND DESIGN

We chose 560 4- to 8-letter words from the MRC Psycholinguistic database (Coltheart, 1981), out of which 224 were used as a source of novel foils in recognition tests. These were paired to create 112 pairs to be included in four simple recognition tests and four recombined recognition tests. The remaining 336 words were randomly paired to create the 168 pairs presented at study. These study pairs were assigned to four different lists of 42 pairs. Each participant studied all four lists of pairs, two in the distraction condition and two in the quiet condition. The assignment of a list to condition was counterbalanced across participants. Pairs of words were presented across the center of a computer screen in 30 point size Times New Roman font.

The pairs in each list were further divided into three sets of 14 pairs each, which were used in three separate recognition tests following each list. In the simple recognition tests, studied pairs were presented alongside foil pairs created from two novel, previously unseen, words. In the associative recognition tests, studied pairs were presented alongside foil pairs created by presenting previously seen words in a new combination. Words for these new combinations were taken from the set of pairs that were used as targets in the same test. For example, if the pairs SLEEP–DAIRY and TABLE–CHURCH had previously been presented, a foil might be SLEEP–CHURCH or TABLE–DAIRY. Thus, in the associative recognition test each studied word was presented twice: once in a target pair and once in a foil pair (see **Figure 2**). In the recombined tests, recombined pairs (e.g., SLEEP–CHURCH) were presented along foil pairs created from two novel, previously unseen, words. The assignment of pairs to the type of test was counterbalanced across participants.

The study conformed to a 2 (distraction condition: silent vs. auditory distraction) × 3 (type of test: simple, associative, recombined recognition) within-participants design. Distraction was manipulated between lists, whereas type of test was manipulated within lists.

Auditory distraction was created by recording words from 18 different semantic categories (Yoon et al., 2004). 15 words, non-overlapping with words presented in the study lists, were taken from each semantic category. The words were spoken in female voice and were recorded at 16- bit resolution and a sampling rate of 44 KHz. The recorded words were combined into two continuous streams of speech, with individual words spoken at the approximate rate of 1 per 750 ms. One of these streams was used for each of the two lists studied and tested in the distraction condition.

### PROCEDURE

Participants studied four lists of pairs, each followed by three recognition tests. The order in which the lists were presented and the order of the pairs within each list was random for each participant. In the study phase, each pair was presented individually for 1500 ms, with 500 ms interval between pairs. Three recognition tests immediately followed the study phase for a given list. Each recognition test (simple, associative, recombined) was preceded by specific instructions, explaining to participants what constituted a target and what constituted a foil in the test. The procedure for all tests consisted of three steps, always administered in the same order. First, target and foil pairs were presented with numbers “1” and “2” (randomly chosen for targets and foils) and a “don’t know” option below each pair. In this free-report step, participants were asked to maximize accuracy and thus indicate a target (by pressing “1” or “2”) only when they were sure which pair is a target, and to respond “don’t know,” by pressing the spacebar, otherwise. Immediately after the response, the pairs were presented again, this time without the “don’t know” option and participants were again asked to indicate which pair they thought they recognized. Finally, the screen was cleared and participants were asked to type in a confidence judgment on a 50 (“guess”)–100 (“sure”) % scale that their response in the forced-report step was correct. The time for responding in all three steps was not limited.

Auditory distraction was played over the noise-canceling headphones during study and test for two lists (the remaining two were studied and tested in silence). Auditory distraction started with the onset of the first study pair in each list and also with the first test pair in each of the three recognition tests. It was however, switched off when participants were reading instructions for each of the tests.

Participants took about 30 min to complete the procedure.

## RESULTS

We organize the result section according to the questions posed in Introduction and referring to (1) memory, (2) resolution of metacognitive monitoring, (3) report criterion, and (4) the IBA and OBA measures of performance. To further disentangle the memory and metamemory effects of distraction on IBA and OBA measures, we also analyze gains of using the “don’t know” option in terms of quality of volunteered responses and losses in terms of quantity of volunteered correct responses. The descriptive statistics can be found in **Table 1**.

**Table 1:** Table showing mean recognition accuracy (hit rate) in the forced-report tests, resolution of metacognitive monitoring (measured by area under the curve, *AUC*), report criterion adopted in free-report tests (measured by the *P*_rc_ measure), output-bound accuracy in the free-report tests (OBA), and input-bound accuracy in the free-report tests (IBA).

	Distraction			Quiet		
	Simple recognition	Associative recognition	Recombined recognition	Simple recognition	Associative recognition	Recombined recognition
Forced-report accuracy	0.78 (0.02)	0.69 (0.02)	0.74 (0.02)	0.83 (0.02)	0.73 (0.03)	0.79 (0.02)
*AUC*	0.73 (0.02)	0.67 (0.03)	0.66 (0.03)	0.78 (0.02)	0.68 (0.02)	0.71 (0.03)
*P*_rc_	66.97 (2.50)	65.26 (2.31)	68.03 (2.65)	66.32 (2.56)	65.53 (2.14)	67.11 (2.52)
OBA	0.90 (0.02)	0.80 (0.03)	0.83 (0.02)	0.92 (0.02)	0.81 (0.03)	0.87 (0.02)
IBA	0.49 (0.03)	0.47 (0.03)	0.44 (0.04)	0.59 (0.03)	0.53 (0.03)	0.53 (0.03)
Gains in OBA	0.12 (0.02)	0.10 (0.02)	0.08 (0.01)	0.09 (0.01)	0.07 (0.01)	0.08 (0.01)
Losses in IBA	0.29 (0.02)	0.22 (0.02)	0.30 (0.03)	0.24 (0.03)	0.20 (0.02)	0.25 (0.02)
Mean confidence	77.12 (1.79)	77.19 (1.69)	71.52 (1.91)	80.70 (1.68)	80.01 (1.73)	77.14 (1.81)
Proportion “don’t know”	0.38 (0.06)	0.35 (0.05)	0.42 (0.07)	0.30 (0.05)	0.29 (0.04)	0.34 (0.05)

*Gains in OBA between forced- and free-report tests, losses in IBA between forced- and free-report tests, confidence judgments provided for correct forced-report recognition decisions and “don’t know” responses for answers correct on the forced-report tests are all proportion measures. Standard errors of the means are given in parentheses.*

### MEMORY

The recognition tests used in the present study were 2AFC tests and thus recognition hit rates in these tests serve as a measure of recognition discrimination. We analyzed hit rates in forced-report recognition tests, which did not allow for withholding responses and thus remained unaffected by any effects distraction could have on metacognitive monitoring and control of retrieval. For completeness, in this and later analyses both partial η^2^and η^2^ are reported as effect sizes. Partial η^2^ is arguably a more appropriate effect-size measure for repeated measures designs because error due to the participant is always included in the denominator when calculating η^2^ (hence partial η^2^ will give a larger effect size estimate than η^2^ for such designs), however, η^2^ is more readily transformed for purposes of meta-analysis and other comparisons across studies. A 2 (distraction: present vs. absent) × 3 (type of test: simple, associative, recombined) repeated measures analysis of variance (ANOVA) on hit rates in forced-report recognition yielded a significant main effect of distraction, *F*(1,41) = 13.85, MSE = 0.01, *p* < 0.001, $\eta_{p}^{2}$ = 0.25, η^2^ = 0.15, by which performance was better when distraction was absent in the quiet condition than when distraction was present. A main effect of test was also significant, *F*(2,82) = 13.18, MSE = 0.02, *p* < 0.001, $\eta_{p}^{2}$ = 0.24, η^2^ = 0.05. This effect arose because, collapsing across distraction conditions, recognition performance was better in the recombined recognition test than in the associative recognition test, *t*(41) = 2.59, *SE* = 0.02, *p* = 0.01, and still better in the simple recognition than in the recombined recognition test, *t*(41) = 2.53, *SE* = 0.02, *p* = 0.02. The interaction of distraction and type of test was not significant, *F* < 1, indicating that distraction disrupted forced responding hit-rates on all types of tests to a similar extent.

Altogether, these results show that auditory distraction negatively affects memory processes, which finds its reflection in impaired memory performance on tests in which participants cannot withhold answers. In this, our results support the earlier finding documenting distraction effects in forced-report tests (e.g., Beaman and Jones, 1997). However, these results do not support the hypothesis that distraction is more harmful to performance on tests based on recollection than tests based on familiarity (see Wais and Gazzaley, 2011). We assumed that performance in the simple recognition test could rely on both familiarity and recollection whereas performance in associative recognition would rely mostly on recollection and performance in recombined recognition would rely mostly on familiarity. The pattern of differences in the level of performance seems at least consistent with these assumptions, with performance in simple recognition (supported by two processes) reliably higher than performance in the remaining two tests (supported by one process). Despite these differences in performance, however, distraction had a similar disruptive effect on all these tests. We return to this observation in the Discussion.

### RESOLUTION OF METACOGNITIVE MONITORING

**FIGURE 3:**
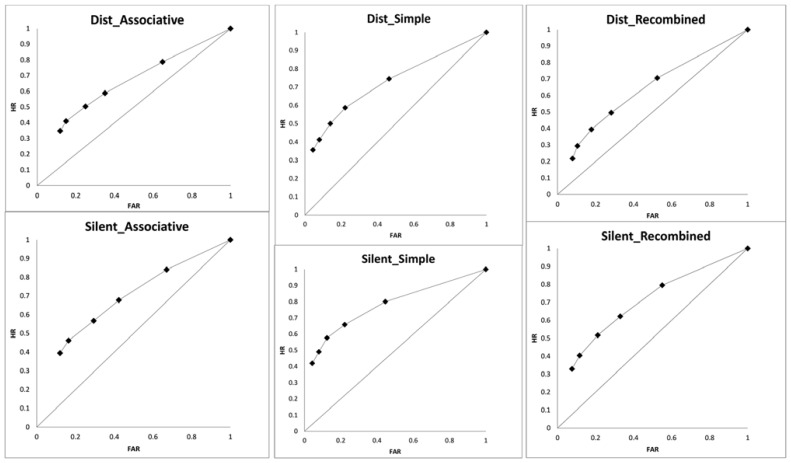
**Hit rates (HR) plotted against false alarm rates (FAR) for distraction (Dist) and silent control conditions on the associative, simple, and recombined testing conditions**.

We turn now to the resolution of metacognitive monitoring, which is participants’ ability to distinguish between their own correct and incorrect candidate responses^1^. Resolution is assessed by examining the relationship between confidence judgments given to responses in forced-report test and correctness of these responses, under the assumption that participants’ metacognitive monitoring is more accurate if they are more confident in their correct responses and less confident in their incorrect responses. Traditionally, gamma correlations have been used to assess this relationship by researchers interested in metacognition (e.g., Koriat and Goldsmith, 1996) although other measures of calibration and resolution (e.g., over/under confidence statistic, point bi-serial, resolution and slope correlation) are available and, in particular, have been used more extensively by researchers interested in the relationship between eyewitness confidence and accuracy (e.g., Juslin et al., 1996; Olsson et al., 1998; Brewer, 2006; Brewer and Wells, 2006; Krug, 2007; Sauer et al., 2010). Recent work in the area of metacognition has also revealed that other measures – those based on signal detection theory – better serve to reveal the accuracy-confidence relationship in these judgments also (Masson and Rotello, 2009; see also Higham, 2002, 2007). Accordingly, we used a non-parametric measure of area under the curve (*AUC*) to assess resolution of metacognitive monitoring as expressed in confidence judgments^2^. A 2 (distraction) × 3 (type of test) ANOVA on the *AUC* measure yielded a significant main effect of distraction, *F*(1,36) = 4.13, MSE = 0.02, *p* = 0.049, $\eta_{p}^{2}$ = 0.10, η^2^ = 0.02, with lower resolution in the presence of distraction. A main effect of type of test was also significant, *F*(2,72) = 8.93, MSE = 0.01, *p* < 0.001, $\eta_{p}^{2}$ = 0.20, η^2^ = 0.08. This effect emerged because, collapsed across distraction conditions, resolution was better in the simple recognition tests as compared to both associative recognition, *t*(36) = 4.14, *SE* = 0.02, *p* < 0.002, and recombined recognition, *t*(36) = 3.31, *SE* = 0.02, *p* = 0.002, while the resolution in the latter tests did not differ, *t* < 1. The interaction of distraction and type of test conditions was not significant, *F* < 1. The curves from which the *AUC* measure was derived are given in **Figure 3**.

The main conclusion from the above analyses is that distraction impairs resolution of metacognitive monitoring. Thus, the distraction seems to be doubly damaging. It undermines memory performance by impairing access to memory records (as described earlier) and it also impairs participants’ ability to indicate which of their responses in memory test are correct and which are incorrect.

### REPORT CRITERION

The third question addressed here is whether in the presence of distraction participants adjust their report criterion. The measure of criterion placement, *P*_rc_, was computed according to the methodology described by Koriat and Goldsmith (1996). Each decimal value on a confidence scale was considered as a possible placement of the report criterion by computing, first, the number of items that were assigned confidence higher than this value and which were volunteered in the free-report test (hits), and second, the number of items that were assigned confidence lower than this value and which were withheld in the free-report test (correct rejections). Hits and correct rejections were used to derive a fit ratio, which is a ratio of the sum of hits and correct rejections to the number of items in a memory test. The value with the highest fit ratio was chosen as the placement of the report criterion and whenever an interval of values was characterized by the same fit ratio, the high boundary of this interval was chosen. This procedure of deriving the *P*_rc_ measure was followed in all six condition of the study. Reduced degrees of freedom for the analyses of *P*_rc_ reflect the fact that some participants either volunteered or withheld all answers in the free-report test in at least one of the conditions, precluding the calculation of *P*_rc_. This measure was analyzed with a 2 (distraction) × 3 (type of test) ANOVA, which failed to reveal any significant effects, *F*(2,74) = 1.13, MSE = 79.66, *p* = 0.33, for the main effect of test, and *F*s < 1, for the main effect of distraction and the interaction. These results indicate that the level of confidence required by participants to volunteer an answer in the free-report test was independent of the lack of distraction and the type of test.

### IBA AND OBA MEASURES OF PERFORMANCE

Finally, we examined the IBA and OBA measures of memory performance in the free-report test^3^. For the OBA measure of performance in free-report tests, a 2 (distraction) × 3 (type of test) ANOVA yielded a significant main of type of test, *F*(2,80) = 15.87, MSE = 0.02, *p* < 0.001, $\eta_{p}^{2}$ = 0.28, η^2^ = 0.19, which exactly paralleled the effect observed for forced-report accuracy: better performance for the recombined test than for the associative test, *t*(40) = 2.33, *SE* = 0.02, *p* = 0.02, and still better performance for the simple (vs. recombined) test, *t*(40) = 3.02, *SE* = 0.02, *p* = 0.004. The main effect of distraction was not significant, *F*(1,40) = 3.22, MSE = 0.01, *p* = 0.08. The same 2 (distraction) × 3 (type of test) ANOVA on the IBA measure yielded a significant main effect of distraction, *F*(1,41) = 27.30, MSE = 0.02, *p* < 0.001, $\eta_{p}^{2}$ = 0.40, η^2^ = 0.12, showing that IBA was worse under distraction. A main effect of type of test came close to significance, *F*(2,82) = 3.06, MSE = 0.02, *p* = 0.052, $\eta_{p}^{2}$ = 0.07, η^2^ = 0.03. This arose because IBA was higher in simple recognition than in associative recognition, a result which also just missed significance, *t*(41) = 1.89, SE = 0.02, *p* = 0.07, and higher also in simple recognition than in recombined recognition, *t*(41) = 2.60, SE = 0.02, *p* = 0.01^4^. Altogether, these results largely track the effects obtained with forced-report accuracy and, most importantly, indicate that participants’ performance, at least if indexed by the IBA measure, is impaired under distraction when participants can respond “don’t know” in a memory test.

### GAINS AND LOSSES FROM USING THE “DON’T KNOW” OPTION

The fact that performance was impaired in free-report tests under distraction can be easily explained by the fact that distraction affects memory access directly, as revealed in the forced-report steps. Interest remains, however, in how metamemory processes contribute to the effects observed in the OBA and IBA measures. To examine this issue, we focused on changes in performance indices between forced- and free-report tests. Koriat and Goldsmith (1996) describe how allowing participants to respond “don’t know” should lead to participants screening out candidate responses they are not sure about, which in turn should generally lead to an increase in the quality of output at the cost to the quantity (see also **Figure 1**). The question addressed in the next set of analyses is whether the increases in quality and reductions in quantity between forced- and free-report tests were influenced by the distraction condition.

First, we calculated increases in quality brought about by exercising control over reporting in free-report tests. For each participant, we subtracted his or her forced-report accuracy from the OBA measure in order to calculate the gains achieved by withholding answers. For this measure, higher scores indicate a larger increase in the quality of output between forced- and free-report tests. The analysis of gains of exercising the “don’t know” option with a 2 (distraction) × 3 (type of test) ANOVA revealed no significant main effect of distraction, albeit a main effect that once again came close to conventional significance, *F*(1,40) = 3.44, MSE = 0.01, *p* = 0.07, $\eta_{p}^{2}$ = 0.079, η^2^ = 0.02. This arose because participants’ quality of output increased more from forced- to free-report tests when distraction was present. Although this analysis comes close to suggesting larger gains from exercising the “don’t know” option under distraction, the compared conditions differ also in the forced-report recognition performance, which was lower under distraction. Any difference in gains may thus be simply due to the fact that more correct responses are to be gained by using the “don’t know” option in the distraction condition. To verify if this is indeed the case, we collapsed across type of test conditions (which did not interact with distraction in the initial analysis) and performed an additional analysis of covariance which controlled for the difference in forced-report recognition performance when comparing gains between two distraction conditions. With the difference in forced-report recognition performance as a covariate, the difference in gains between distraction and silent conditions fell well short of conventional significance, *F* < 1. It thus appears that although participants gain more in terms of quality when they use the “don’t know” option under distraction, this effect can be accounted for simply by the greater potential for gain given the lower baseline rather than any more fundamental difference in the effectiveness of metacognitive processes.

To construct the measure of the reduction in the quantity of output, we subtracted the IBA measure from forced-report accuracy. For this measure, higher scores mean that more correct responses were lost from forced- to free-report test. The analysis of losses from exercising the “don’t know” option with a 2 (distraction) × 3 (type of test) ANOVA revealed a significant main effect of type of test, *F*(2,82) = 12.37, MSE = 0.02, *p* < 0.001, $\eta_{p}^{2}$ = 0.23, η^2^ = 0.11, which arose because losses were reliably lower in the associative recognition test, than in both simple recognition, *t*(41) = 3.87, SE = 0.01, *p* < 0.001, and recombined tests, *t*(41) = 5.13, SE = 0.01, *p* < 0.001. Losses for the latter two tests did not differ from each other, *t* < 1. More importantly, a significant main effect of distraction was revealed, *F*(1,41) = 9.74, MSE = 0.01, *p* = 0.003, $\eta_{p}^{2}$ = 0.19, η^2^ = 0.05, which points to higher losses from exercising the “don’t know” option in the presence (vs. absence) of distraction. The interaction was not significant, *F* < 1. We conducted a similar covariance analysis as before to account for the difference in the forced-report recognition performance between distraction and silent conditions. Collapsing across type of test conditions, the analysis with the difference in forced-report performance between silent and distraction conditions as a covariate still revealed a reliable difference between distraction and silent conditions in terms of losses, *F*(1,40) = 10.34, MSE = 0.003, *p* = 0.003, $\eta_{p}^{2}$ = 0.21, η^2^ = 0.20. Thus, in contrast to the analysis of gains, greater losses in the quantity of output in the distraction condition were not due to differences in the forced-report recognition performance between silent and distraction conditions.

The analyses of gains and losses arising from exercising the “don’t know” option revealed that allowing the “don’t know” response in a memory test under distraction bears important consequences for the final performance. On the one hand, participants in the present study initially appeared to gain more in terms of quality in the distraction compared to the silent condition. This effect, however, did not stem from any differences in metacognitive processes but simply from the fact that with lower memory performance participants had more to gain under auditory distraction. More cogently, the presence of distraction caused also greater losses in term of quantity of correct responses when participants were allowed to respond “don’t know.” This effect was independent of differences in forced-report recognition in silent and distraction condition and thus must reflect a metacognitive effect.

The effect of greater losses of correct responses under distraction might, in principle, be the result of two different mechanisms. One such mechanism is reduced relative accuracy of metacognitive monitoring under distraction. When participants are worse in assessing which their candidate responses are correct and incorrect, using the “don’t know” option can lead to withholding of more correct responses. To assess this account, we performed an additional covariance analysis in which differences in the *AUC* measure between the distraction and silent conditions (again collapsed across different tests) served as a covariate for the analysis of losses^5^. This analysis again resulted in a reliable differences in losses between the distraction and silent conditions, *F*(1,35) = 4.79, MSE = 0.003, *p* = 0.035, $\eta_{p}^{2}$ = 0.12, η^2^ = 0.11, suggesting that differences in the accuracy of metacognitive monitoring cannot account for the observed pattern of losses.

A second mechanism that can account for greater losses under distraction is reduced confidence in correct responses. If participants are overall less confident in their correct responses under distraction, then fewer of these correct candidate responses will pass the rigid response criterion (see the earlier analysis of the *P*_rc_ measure). To test this hypothesis we analyzed confidence for answers that were correct on the forced-report recognition tests. The analysis of confidence with a 2 (distraction) × 3 (type of test) ANOVA yielded a significant main effect of test, *F*(2,82) = 12.45, MSE = 44.21, *p* < 0.001, $\eta_{p}^{2}$ = 0.233, η^2^ = 0.114, which arose because confidence in correct responses was lower in the recombined test than in either the simple recognition test, *t*(41) = 4.33, SE = 1.06, *p* < 0.001, or the associative recognition test, *t*(41) = 3.87, SE = 1.10, *p* < 0.001, which did not differ from each other, *t* < 1. More importantly, the main effect of distraction was also significant, *F*(1,41) = 26.97, MSE = 37.50, *p* < 0.001, $\eta_{p}^{2}$ = 0.397, η^2^ = 0.105, confirming that participants were less confident in their correct responses under distraction. The interaction was not significant, *F*(2,82) = 1.58, MSE = 28.01, *p* = 0.21. To ensure that lowered confidence in correct responses under distraction found its reflection in the pattern of response volunteering, we also analyzed the rates of “don’t know” responding for responses that were correct on the forced-report recognition test. A 2 (distraction) × 3 (type of test) ANOVA yielded a significant main effect of type of test, *F*(2,82) = 4.99, MSE = 0.017, *p* = 0.009, $\eta_{p}^{2}$ = 0.108, η^2^ = 0.045, which arose because participants more often responded “don’t know” for questions scored as correct in the forced-report recombined test than in either the forced-report simple recognition test, *t*(41) = 2.06, SE = 0.02, *p* = 0.046, or the forced-report associative recognition test, *t*(41) = 2.88, SE = 0.02, *p* = 0.006, which in turn did not differ, *t*(41) = 1.14, SE = 0.02, *p* = 0.26. Again, the main effect of distraction was significant, *F*(1, 41) = 15.94, MSE = 0.02, *p* < 0.001, $\eta_{p}^{2}$ = 0.280, η^2^ = 0.09, with fewer responses that were correct on the forced-report test volunteered under distraction^6^. These analyses indicate that distraction affects performance when a “don’t know” response is allowed by reducing confidence in correct candidate responses, which leads to fewer correct responses being volunteered. Overall, reduced confidence under distraction is related to greater response withholding, this constitutes a metacognitive mechanism by which distraction exerts influence over memory performance.

## DISCUSSION

The present study was designed to investigate memory and metacognitive processes under auditory distraction. The main results can be summarized in few points. First, distraction consistently impaired memory as revealed by forced-report recognition accuracy. Thus, there was an effect of distraction on cognition under forced-choice conditions. Second, distraction affected metacognitive monitoring of retrieval by impairing participants’ ability to distinguish between their own correct and incorrect candidate responses (as reflected in the confidence measures). Thus, there was also an effect of distraction on metacognitive outcomes. Third, participants did not try to strategically compensate for the loss in the quantity of output under distraction by lowering their report criterion. Instead, participants used the same report criterion in all conditions of our study. Fourth, distraction affected both benefits and costs of using the “don’t know” option. Larger gains in accuracy under distraction stemmed from the fact that, with poorer memory in the presence of distraction, participants had more to gain by withholding responses. However, losses in terms of quantity of correct responses were also larger under distraction, stemming from the fact that participants were generally less confident in their correct responses when distraction was present, leading to more prevalent withholding of these correct responses. Thus, there appeared to be an impact of some metacognitive factors (confidence) but not on others (report criterion or threshold) on the distraction observed. A limitation of the study is that, because distraction was present at both encoding and retrieval we are unable to tease apart potentially different impacts on metacognitive processes at these stages. The literature on auditory distraction shows that some distractors (so-called “changing-state” irrelevant sound distractors, Jones et al., 1992; Jones and Macken, 1993) operating on short-term serial order episodic memory (Beaman and Jones, 1997) are equipotent at encoding and retrieval (Miles et al., 1991) whereas semantic auditory distractors operating on semantic memory lose potency to induce false positive errors when presented only during a retention period (Marsh et al., 2008). Thus, this particular area is open to further research in which both the types of distraction and the period of distraction are varied.

The fact that auditory distraction is harmful for visual memory performance is hardly surprising given numerous studies which already document such detrimental effects. However, the present study allows for a clearer picture of how impairment in performance is caused by distraction affecting both memory access directly, and metacognitive processes responsible for translating retrieved information into free-report performance. The study was built on the assumption borrowed from the framework by Koriat and Goldsmith (1996) that performance in a test allowing for “don’t know” responses is dependent on both memory and metacognitive processes. Here we demonstrated that auditory distraction affects both memory and metacognition, which jointly determine performance in free-report tests.

In our study we included a forced-report test in which withholding responses was not possible, thus minimizing the role of metacognitive processes. The fact that distraction impaired forced-report responding provides further support for previous research using this format of testing (e.g., LeCompte, 1994; Beaman and Jones, 1997) in showing that auditory distraction has a negative impact on core memory processes. Importantly, we used three different types of recognition test, in which performance was at least partially supported by distinct memory processes of familiarity and recollection and yet the effects of auditory distraction were exactly the same in all these tests. This observation points to a general disruptive effects of distraction upon various memory processes regardless of the relative contribution of recollection and familiarity in these tests. This is surprising given the differential impact of auditory distraction upon other memory tasks – notably the reliable impact upon probed or serial recall which is either absent or much reduced in a “missing item task” in which participants are required to identify which member of a well-known set was not presented at study (Beaman and Jones, 1997). Here, it seems plausible that familiarity might be used to identify missing items (by recalling all items from the set and assessing which has the lowest familiarity level) whereas recollection is necessary for probed recall. These tasks differ in other ways, however, notably the requirement to recall a pairwise, positional, or serial-order association for the probed recall task – arguably also present in the current set of 2AFC recognition tests, which is entirely absent from a missing item recall task (see also Jones and Macken, 1993; Beaman and Jones, 1998).

In this observation of generalized effects of auditory distraction by speech on memory processes, our results also stand in contrast to the recent results obtained by Wais and Gazzaley (2011), who documented the effects of distraction in a test dependent on recollection but not in the test dependent on familiarity. Various procedural differences may underlie this discrepancy but the most likely seems to be that whereas Wais and Gazzaley (2011) played distraction during retrieval only, in our study distraction was present during both encoding and retrieval. Some researchers (e.g., Neath, 2000) have argued that the effects of auditory distraction are similar to the effects of manipulations imposing cognitive load, like for example requiring participants to engage in an additional cognitive task apart from encoding and retrieval. Dual-task manipulations, when implemented at the time of a memory test, are known to affect recollection, leaving familiarity relatively intact (e.g., Gruppuso et al., 1997). By contrast, dual-task manipulations implemented at the time of encoding affect both recollection and familiarity (Yonelinas, 2001). Thus, our findings of auditory distraction effects of similar magnitude in recollection- and familiarity-based tests can be easily reconciled with findings pointing to specific recollection impairment obtained by Wais and Gazzaley (2011) if one is willing to assume that auditory distraction, in some respects, works similarly to an additional cognitive task, putting tax on attentional resources available for encoding and retrieval^7^. Pilot data from our lab support this idea, by showing an impact of auditory distraction upon pupillometric measures of cognitive effort at encoding. To conclude, it is likely that it is our design choice of presenting distraction both at encoding and at retrieval that precluded us from observing differential effects of auditory distraction on familiarity and recollection. This issue could be pursued in further studies that could factorially manipulate when distraction is presented to examine performance in recognition tests sensitive to familiarity and recollection effects.

The results documented in our study with free-report tests also reveal that effects of distraction do not end with impairing memory processes. Auditory distraction has important consequences for how accurate people are in monitoring their memory processes, as revealed by impaired resolution of confidence judgments under distraction. Even more importantly, auditory distraction modifies metacognitive control and thus shapes performance when the “don’t know” option is available in a memory test. Participants seem to be aware that auditory distraction is harmful for memory as they become much less confident in their correct responses when distraction is present (see also Ellermeier and Zimmer, 1997; Beaman, 2005b). In their responding on a free-report test they strive to attain a similar level of accuracy of reported responses whether distraction is present or not, as revealed by an equal report criterion between distraction and quiet conditions. However, since participants are generally less confident, fewer correct candidate responses pass the report criterion when distraction is present. With fewer correct responses volunteered, the IBA measure of accuracy suffers. Not only is the IBA measure lower under distraction but also losses in quantity caused by using the “don’t know” option are higher when distraction is present.

This last finding is important inasmuch as it testifies to metacognitive contributions to performance decrement caused by distraction in free-report tests. When free-report test are used, the IBA measure of performance is commonly interpreted as reflecting memory processes only (cf. Koriat and Goldsmith, 1996; Hanczakowski et al., 2014). This can be gleaned from paradigms using free recall, a par excellence IBA measure of performance, in which results are discussed in terms of memory, not metamemory. In respect to auditory distraction, several recent papers dealing with distraction semantically related to memoranda have revealed performance decrements in free recall tests under semantic auditory distraction (e.g., Marsh et al., 2008, 2009). What the present study underscores is that results obtained with free recall tests should be interpreted in view of distraction impacting upon both memory and metacognitive processes. It thus remains to be examined if auditory distractions semantically related to memoranda impact upon metacognitive processes, changing the pattern of confidence and “don’t know” responding, and thus contributing to the overall pattern of free recall impairment.

In conclusion, the present study showed how auditory distraction affects both memory processes and metacognitive processes that influence memory reporting. In broadest terms, auditory distraction when present at both encoding and retrieval negatively impacts upon a spectrum of performance measures in memory tasks. However, a specific pattern of impairment in these measures is visible, shaped by the effects distraction has on metacognitive processes, with important roles of the overall level of confidence assigned to correct candidate responses and the ability to distinguish between one’s own correct and incorrect candidate responses.

## Conflict of Interest Statement

The authors declare that the research was conducted in the absence of any commercial or financial relationships that could be construed as a potential conflict of interest.

## Footnotes

1 Resolution is one aspect of the accuracy of metacognitive monitoring. The other aspect is referred to as calibration. Whereas resolution computed based on confidence judgments captures the degree to which correct candidate responses receive higher judgments than incorrect responses, calibration measures capture the degree to which mean of confidence judgments correspond to the mean memory performance. Calibration scores are usually interpreted in terms of realism of metacognitive monitoring: when mean confidence judgments are lower than memory performance participants are said to be underconfident and when mean confidence judgments are higher than memory performance participants are said to be overconfident. However, the psychological interpretations of calibration measures have recently been questioned based on the observation that calibration scores derived from confidence judgments do not chime with the results derived from other tasks, like binary judgments or betting decisions (Hanczakowski etal., 2013; Higham etal., in press; see also Scheck and Nelson, 2005; England and Serra, 2012, for related discussion). Given the theoretical problems surrounding calibration results, we did not analyze this aspect of the accuracy of metacognitive monitoring in the present study.

2 The degrees of freedom in this analysis differ from other analyses presented here because the *AUC* measure in at least one of the condition could not be computed for five participants, who had no incorrect responses in some conditions. These participants were excluded from this analysis.

3 One participant was removed from all analyses involving the OBA measure because this person responded “don’t know” to all questions in one of the conditions, precluding the calculation of the OBA measure.

4 According to the yes/no logic of significance testing, these results should be treated with caution as there is an enhanced possibility of false positives when reporting such results after a null main effect. However, it is of interest to see how the data break down in cases (such as this) which so narrowly fall short of reaching conventional significance and we do not base any strong theoretical claims on these particular outcomes.

5 Five participants were excluded from this analysis due to incomplete set of *AUC* results.

6 Although incorrect responses do not contribute to the pattern of losses which is discussed here, for completeness we separately analyzed confidence and the rate of “don’t know” responses when an incorrect response was given in the forced-report test. Since quite a few participants had no incorrect forced-report responses in one or more cells of the full design, for the present analyses we collapsed across the type of test variable, which resulted in excluding two participants only. The comparisons of quiet and distraction conditions in terms of both confidence in incorrect forced-report responses (62.14 vs. 63.15) and the rate of “don’t know” responses when forced-response was incorrect (0.66 vs. 0.66) were not significant, *t*s < 1.

7 Note, however, that there are multiple types of auditory distraction (e.g., those based around the acoustics of the distractor and those based upon the lexical and semantic status of speech) which appear to impact on different tasks differentially (Hughes and Jones, 2003; Beaman, 2005a; Jones etal., 2010). Without further investigation varying the distractor type do not know which type of distraction we (or Wais and Gazzaley, 2011) observed.
